# Music perception and movement deterioration in Huntington’s disease

**DOI:** 10.1371/currents.RRN1252

**Published:** 2011-09-20

**Authors:** Christian Beste, Anne Schüttke, Bettina Pfleiderer, Carsten Saft

**Affiliations:** ^*^Institute for Cognitive Neuroscience, Ruhr-University, Bochum, Germany; ^†^Department of Clinical Radiology, University of Münster, Germany and ^§^Department of Neurology, Ruhr-University, St. Josef-Hospital, Bochum, Germany

## Abstract

Background: There is increasing evidence for functional interactions of the auditory and the motor system in music perception. Based on that we hypothesized that altered music perception in patients with a movement disorder, here Huntington’s disease (HD), compared to controls should be present. Additionally, there should be also a relation between areas associated with the assessment of musical rhythms and measures of movement deterioration in patients.

Methods: Manifest (HD) and premanifest HD (pHD), as well as healthy controls underwent an examination with auditory functional MRI (fMRI) with presentation of music and syllables as stimuli. Additionally, motor performance was assessed in tasks with different complexity and related to fMRI-data.

Results: There was a significant interaction of type of stimuli (music, syllables) and group (HD, phD, controls). During music processing when compared to blocks with syllables only, HDs revealed hyperactivations, especially in cerebellar structures,. In contrast, these structures were stronger activated during syllable presentation in pHD´s and controls, when compared to HD and music processing. Increased cerebellar activations during music processing in HDs were related to more severe voluntary and involuntary movement dysfunction. No correlations were observed with activations after syllable presentation. Generally, no relations were found in pHDs.

Conclusion: The results suggest modulation of auditory music processing in a movement disorder, which seems to relate to severity of movement deterioration.

## **Introduction**

Huntington’s disease (HD) is an autosomal, dominant inherited neuropsychiatric disorder typically characterized by chorea and involuntary movements. Genetically, HD is caused by an extension of the CAG-repeat length at the 4^th^ chromosome encoding a large protein, the huntingtin, which accumulates and causes apoptotic striatal neuronal death.^[1]^ Neuroanatomical pathology is not limited to the striatum but is also seen in other brain regions like the thalamus as well as parts of the neocortex.^[2][3][4]^ General motor impairment, which are assessed via the Unified Huntington’s Disease Rating Scale (UHDRS) motor score, includes impairment of involuntary and voluntary movements and can be measured in various motor tasks differing in complexity.^[5][6][7][8]^ In the latter, manifest patients present progressive deterioration during course of disease.^[9][10][11][12]^

Motor performance is associated with several other “cognitive” processes, too. Recently it has been shown that it is influenced by music. For example, Lahav et al. reported that motor performance can be enhanced even by passive listening to music.^[13]^Other studies described functional overlaps between perception and production of music suggesting a close interaction of the auditory and the motor system in music perception and production.^[14][15][16][17][18]^

For the processing of music and especially for rhythm and beat perception cerebellar structures are particularly of importance.^[19][20][21][22]^ These structures are affected in HD and it has been shown that cerebellar dysfunctions contribute to cognitive symptoms in HD.^[8][23][24]^ Assuming a close relationship between motor functions and music perception, one may hypothesize (i) that patients with HD will show altered processing of music that depends on disease stages, i.e. between manifest HD and premanifest HD (pHD) and (ii) that there should also be a relation between areas associated with the assessment of musical rhythms (cerebellar structures) and measures of voluntary movement deterioration in patients. As a control condition blocks of syllables were chosen and we expected these changes not to occur while listening to syllables.   

## **Materials and Methods**

### ***Participants***

Fifteen patients with genetically confirmed and clinically manifest HD were included. Additionally 16 premanifest gene mutation carriers, defined by a positive gene test and absence of unequivocal motor symptoms were recruited (UHDRS diagnostic confidence level 0 to 3).^[5]^ All HD and pHD participants were right handed and underwent extensive neurological investigation and were scored according to the motor scale (MS), total functional capacity (TFC) and independence scale (IS) of the unified Huntington’s disease rating scale. Cognitive performance was evaluated by the verbal fluency test, symbol digit test and stroop interference test. The results of these tests were summarized as “cognitive score” (CS; UHDRS).^[5]^ Fine motor skills were measured by simple (tapping; higher motor impairment leads to lower test results) and complex (peg board; higher motor impairment leads to higher test results) instrumental movement tests.^[6][7]^ Detailed patient data are given in table 1.

Separate age and gender matched volunteers were recruited, serving as controls for HD (N = 15; age = 41.5 ± 9.25) or pHD patients (N = 16; age = 34.5 ± 5.5). All participants gave written informed consent. The study was approved by the ethics committee of the Ruhr-University of Bochum (Vote Nr. 2485).    

**Table:** 

	**HD**	** pHD**	**Significance**
**Age**	42.13(12.47)	36.31 (9.63)	ns
**sex (male/female)**	11/4	7/9	-
**CAG-length**	45.5 (4.98)	42.62 (1.7)	p < .05
**estimated age of onset (eAO)**	-	52.82 (7.41)	-
**age of onset (AO)**	40.26 (13.74)	-	-
**duration of disease (years)**	1.86 (3.9)	-	-
**UHDRS MS**	26.66 (12.95)	1.94 (1.30)	p < .001
**Tapping**	141.90 (34.29)	176.21 (20.51)	p < .001
**Peg board**	527.36 (122.5)	397.37 (30.86)	p < .001
**UHDRS CS**	152.7 (44.56)	229.68 (29.83)	p < .001
**UHDRS TFC**	11.73 (1.09)	12.93 (0.25)	p < .001
**UHDRS IS **	88.83 (7.94)	99.37 (2.5)	p < .001
**Beck Depression Inventory**	6.06 (5.61)	6.93 (7.049	ns
**Hamilton Depression Scale **	8.4 (6.1)	5.5 (6.4)	ns

Table 1: Clinical characteristics of manifest HD and premanifest HD (pHD). Values are given as mean and standard deviation in brackets. Abbreviations: AO, age at symptom onset; eAO, estimated AO, YTO, years to estimated AO; UHDRS, unified Huntington’s disease rating scale; IS, independence scale; TFC, total functional capacity; MS, motor score; CS, cognitive score, for tapping and peg board values performance was collapsed over the left and right hand. 

### ***Experimental Design***

The complete auditory stimulation paradigm consisted of three different types of stimulation blocks: A1–A3 (music (A1M; recording of a piano piece of Alkan: Barcarole), sine wave tones (A2T; digitally generated pulsed (frequency 5 Hz) 800 Hz) and a repetitive presentation of the syllable “bla” (bla:-bla ) 1 syllable/ sec., spoken by a male voice,“) (A3S)) of 115 s duration each (ON), alternating with rest periods R1–R4 of 69 s (OFF), respectively (Fig. 1).

**Figure:**
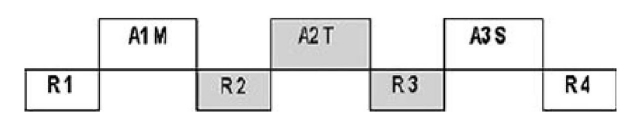

Figure 1: Auditory stimulation paradigm. R1-R4 = rest periods of 69s (OFF). Music (A1M), sine wave tones (A2T) and syllables (A3S) of 115s duration each (ON).

The order of the stimuli was determined after a series of pilot experiments in healthy subjects, which revealed that this sequence yielded the most robust and reproducible activations for all acoustic stimuli. The paradigm was created with the Cool Edit Software program (Syntillium Software Corporation, Phoenix, AZ, USA). Auditory stimulation was presented binaurally via pneumatic headphones (tube and headphones were acoustically shielded to reduce outside and gradient noise). For all subjects, the hearing threshold was determined within the magnet, and each subject was stimulated with a sound pressure level of 85 dB above the individual hearing threshold. In this paper, which is part of a larger study protocol, only the results of music and syllables will be presented because we had already reported results of sine tone stimulation in HD.^[25]^

### ***Assessment of voluntary movements***

We used two motor tasks, one simple (tapping) and one complex task (peg board). In the tapping task subjects were instructed to tap as quickly as possible on a computer-based contact board (3 x 3cm) with a contact pencil for a period of 32 seconds after the initial flash of a yellow stimulus light. The board was positioned in the centre before the patient. When performing the task, elbows were allowed to be in contact with the table. The number of contacts was measured. All subjects underwent familiarization with this task.^[6]^ For statistical analysis performance in this task, measured as the absolute frequency of taps was collapsed over the left and right hand.^[26]^

In the more complex task (peg board) subjects were instructed to transfer 25 pegs (diameter 2.5mm, length 5cm) from a rack into one of 25 holes (diameter 2.8mm) in a computer based contact board individually and as quickly as possible. The distance between rack and appropriate holes was 32cm. The board was positioned in the centre before the patient and the task was carried out with the left and right hand. When transferring each peg from rack to hole, elbows were allowed to be in contact with the table. The time interval between inserting of the first and the last pin was measured. The time period, was assessed by a computer to 100ms accuracy. For statistical analysis, performance was collapsed over the left and right hand. All subjects underwent familiarization with this task.^[7]^    

### ***MRI data acquisition***

MRI data were acquired in a 3 Tesla whole body scanner (Gyroscan Intera T30, Philips, Best, NL) with a circularly polarized transmit/receive birdcage head coil. 36 T1-weighted anatomical spin-echo images (TR = 480 ms, TE = 15 ms, flip angle 90^o^, matrix dimensions: 256 x 256 and field of view (FOV) = 210 mm, slice thickness 3.6 mm) were acquired before the fMRI data sets. For fMRI, 60 gradient echo-planar imaging (GE-EPI) images in identical localization were obtained (TR = 11.5 sec, TE = 60 ms, matrix dimensions: 64 X 64, FOV = 210 mm, slice thickness = 3.6 mm: in-plane resolution 3.6 x 3.6 mm, 36 transversal slices orientated parallel to the anterior commissure – posterior commissure (AC-PC) line, scan time = 3.4 sec). A TR of 11.5 sec was used: “sparse” imaging: auditory stimuli are presented without interfering with scanner noise in long interscan intervals to overcome the confounding effects of scanner noise on the experiments.^[25]^ To reduce movement artefacts subjects were fixed by a tape around his/ her forehead inside the coil.    

### ***fMRI data analysis***

Image processing and statistical analysis of the fMRI images were done by SPM5 standard routines and templates (www.fil.ion.ucl.ac.uk/spm). Images were realigned, normalized and re-sliced to a voxel size of 2x2x2 mm^3^, and smoothed with an 8 mm kernel. Data were filtered with a high-pass filter (cut-off period of 128 s).    

***Cluster and second level analysis***

Here, the activation differences between HD and controls, pHD and controls, HD and pHD were analysed (second level t-test, SPM5, p uncorr. < 0.001, minimum cluster size, k = 10 voxels) and standard stereotaxic coordinates of activated brain regions were determined.^[27]^ To obtain activation maps for either patients (HD, pHD) or controls the functional data were combined in a random-effects analysis to identify activated brain areas (uncorrected for multiple comparisons, *p * < 0.001, extent threshold/minimum cluster size *k * = 10 voxels, one sample *t*-tests). Contrasts that were found to be significant in the random-effect analysis constituted the basis for further hypothesis-directed analysis with the activation intensities in the activated areas.

In a second-level analysis fMRI-data were analyzed in a full-factorial design including stimuli (music or syllable) as within-subject factor and group (HD, pHD, manifest control, premanifest control) as between-subject factor. Activated areas were superimposed on anatomical images for an anatomic localization.

A data-driven ROI analysis, based on the results of the second-level analysis was performed. Here, three cerebellar ROIs, two which had been more active in HDs during music perception (0,-54,-14; 0,-36,-12) and one during syllable processing (-4,-44,6) were selected. Then, a sphere with a radius of 4mm around these activation maxima were used and applied to each subject. For each subject the blood oxygen level-dependent (BOLD) signal intensity (defined by the corresponding weighted *β*-values) was determined. Bonferroni-corrected Pearson correlation analyses with results of the motor performance tasks in the HD and pHD group (tapping, peg board and UHDRS motor score) were calculated using the activation intensities obtained from these ROIs.   

## **Results**

### ***fMRI-data***

There was a main effect of type of stimuli (F(1,116) = 11.39; p < .001 uncorr.). Post hoc tests revealed that music presentation induced higher activity in the parahippocampal gyrus (-36,-46,-4) (BA19) and in the posterior cingulate (12,-28,24), (BA23) compared to syllables.

Additonally there was a main effect of group (F(3,116) = 5.79; p < .001 uncorr). Post-hoc tests indicated lower activations in HD-group compared to pHDs in the right middle temporal gyrus (54,-32,-10) (BA21), left (46,-48,18) (BA31) and right superior temporal gyrus (-42,-54,20) (BA39) and the mediodorsal thalamic nucleus (4,-18,4). When HDs were compared to controls, group differences were due to smaller activations in the left parahippocampal gyrus (-32,-30,-14) (BA36) and the right fusiform gyrus (34,-52,-6) (BA37) in the HD-group. The pHD-group presented higher activations in the right inferior frontal gyrus (55,6,22) (BA44), right fusiform gyrus ((48,-32,-14; BA20) (38,-64,-8; BA19)).

There was a significant interaction “stimuli x group” F(3,116) = 5.79; p = .001 uncorr.) for the left superior frontal gyrus (-10,60,24) (BA10), the cerebellar anterior lobe (0,-36,-8), the right superior temporal gyrus (36,10-22) (BA38) (see Figure 2A) and the mediodorsal thalamic nucleus.     

**Figure:**
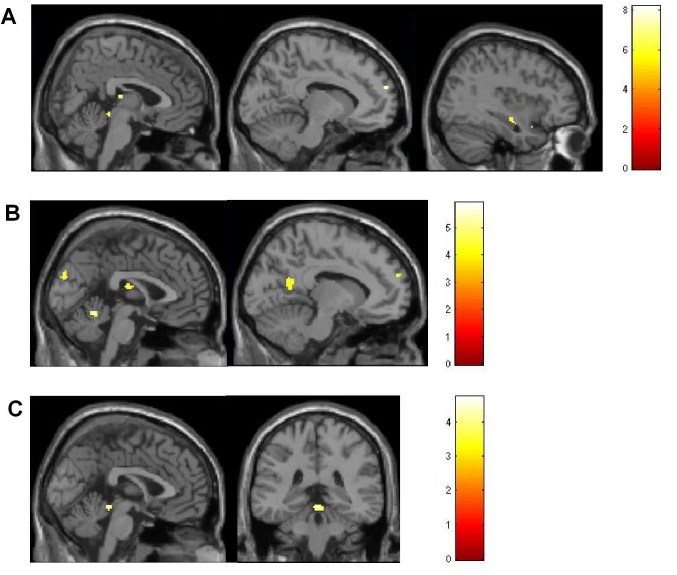

Figure 2: (A) Interaction of “stimuli” (music/syllable) and “group” (HD, pHD, HD-controls, pHD-controls). This interaction can be observed for the left superior frontal cortex (SFG), right superior temporal cortex (STC), the mediodorsal thalamic nucleus and the cerebellar anterior lobe. (B) Areas of higher activation during music compared to syllable processing in HDs. These areas include the right SFG, the posterior cingulate, the mediodorsal thalamic nuclei, the cuneus and the cerebellar culmen. (C) Comparison of the HDs and pHDs during music processing: HDs presented higher activation in the anterior cerebellar lobe during music listening, as compared to pHDs.

Subsequent analyses were performed to explore this interaction in more detail. The HD-group showed higher activations for music vs. syllables presentation (Figure 2B): in the left superior frontal gyrus (-8,58,24), right mediodorsal thalamic nucleus (2,-16,16), the left posterior cingulate (-10,-58,14) (BA30), the left cuneus (-2,-88,24) (BA18) and the culmen (0,-54,-14). No effects were seen using the opposite contrast. For controls and pHD´s the pattern was the opposite. Here, syllables produced higher activations in the left anterior cingulate cortex (ACC) (-14,44,6) (BA32) and right middle frontal gyrus (30,48,10) (BA10) for the controls. For the pHD-group no activity differences were seen between music and syllable perception.

Additionally we found that the cerebellar culmen (0,-36,-12) was more active during music presentation in HDs compared to pHDs (Figure 2C). In contrast, pHDs showed higher activation in the right middle temporal gyrus (42,-60,24) during music presentation, compared to HDs. For the syllable stimuli the HD-group presented smaller acitivations in the right parahippocampal gyrus (18,-32,-10) (BA35) and left cerebellar culmen (-4,-44,-6) compared to pHDs and in thalamic (2,-20,2; 24,-28,4) nuclei as well as in the right parahippocampal gyrus (28,-56,-2) (BA19) and right superior frontal gyrus (2,38,52) (BA8) compared to controls.

### ***Correlational analysis: motor data***

Voluntary movement performance differed between HD-groups, as assessed by the tapping and peg board tests (see Table 1). Pearson correlations were calculated between performances in tapping activity, peg insertation time and activation intensities the cerebellar region in HDs and pHDs. This region was more active in HDs during music presentation (Fig.2b). Performance in the tapping task was inversely related to the activation strengths: the better the motor performance (i.e. more taps) the less active were these cerebellar structures during music listening (see: Figure 3 top). In contrast, peg board performance was positively associated activation intensity in the cerebellum: the better motor performance here (i.e. faster peg insertion), the less active. (see: Figure 3 middle). Remarkably, we found also a correlation with general motor dysfunction as depicted in the UHDRS motor score: higher UHDRS motor scores (i.e. more severe motor symptoms) were associated with increased cerebellar activation during music pereception (see: Figure 3 bottom). No relations regarding tapping or peg insertion (all r’s < .237; p > .189). were observed for the pHD-group (all r’s < .237; p > .189). It is important to note, that even though cerebellar structures were also active during syllable perception in HDs, activation strengths was not correlated with performance in tapping, peg insertion or the UHDRS motor score (see: Figure 3 right column). Also for the other brain regions, where HDs revealed higher activation during music processing, no relations were found. Cerebellar activations were also not related to cognitive deteriorations, as the non-significant correlations with the UHDRS cognitive score (all r’s < .189; p > .455).     

**Figure:**
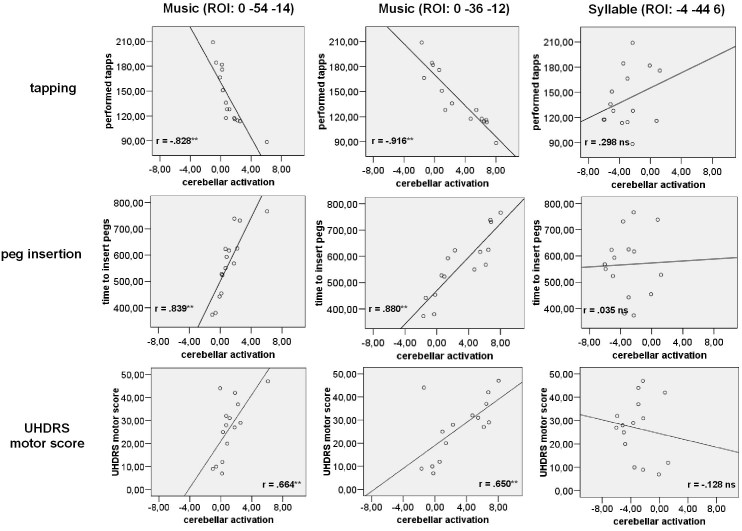

Figure 3: (top) Strong relation can be seen between tapping performance and activation intensities during music perception in cerebellar areas, whereas no relation were found during syllable presentation. (middle). Strong relation was found between peg board performance and activation during music processing in cerebellar areas, whereas no relations were found during syllable presentation. (bottom) A similar correlation pattern was observed for the UHDRS motor score.   

## **Discussion**

In this study we tested the hypothesis of altered music perception in patients with HD – depending on the severity of motor symptoms – and in comparison to controls. Additionally, we postulated that there should be a correlation between areas associated with the assessment of musical rhythms and measures of voluntary movement deterioration in patients, since it was reported previously that there is a strong interaction of the auditory and the motor system in music perception.^[14][15][16][17][18]^ The main results of this study are in line with these assumptions.

We observed an altered activation pattern in manifest HDs compared to premanifest mutation carriers  and controls. HDs revealed overall weaker activations in the right middle temporal gyrus, right superior temporal gyrus (SFG) and the mediodorsal thalamic nucleus, when analysing the group effect across conditions.

Middle temporal areas have been shown to be important for various aspects of auditory processing and especially in sensory gating functions.^[28]^ Parahippocampal regions, which have also been found to be lower activated in HDs compared to controls, are known to be functionally closely related to middle temporal areas and also seem to mediate sensory gating functions.^[29][30]^ This further supports the presence of a sensory gating deficit in HD.^[25][26]^ The overall lower activation (across conditions) in the right SFG and mediodorsal thalamic nuclei in HDs may suggest for the presence of deficits in the attentional network during processing of auditory stimuli.^[31][32]^

One interesting finding of this study, as reflected in the significant interaction of group x stimuli was, that when analysing activations during music perception alone, the results were different. Here we found an increased activation during music listening in manifest HD in cerebellar structures (Fig. 2C) compared to pHDs and controls. Additionally, the SFG, the posterior cingulate cortex and the medio-dorsal thalamic nucleus were hyperactivated in HDs, when comparing music to syllable processing (Figure 2B). This suggests for an increased engagement of attentional networks in HDs during listening to music to extract the complex musical structure.^[31][32][33]^

The finding of an increased activation of cerebellar structures may reflect an increased engagement of these networks required for the processing of the complex rhythmic structure of the music.^[20][21][22]^ This is further underlined by the finding that syllables, where no rhythmic structure was present, did not induce cerebellar hyperactivation in HDs, when compared to music. A comparable hyperactivation has recently been described by Saft et al. for basal ganglia structures (putamen) during sine tone perception in HD only.^[25]^

As pHD-patients presented smaller activations of cerebellar structures during music processing this increased engagement is likely to be part of pathogenic mechanisms. This is even more feasible to assume, since more deficient motor performance as it is usually found in HD, was related to more activated cerebellar structures in this study (Fig. 3).^[6][7]^ This result of a relation of the intensity of activation of the cerebellum during music listening and various motor performance measures may be explained by the presence of overlapping neural networks for music processing and movements as previously suggested.^[18]^ This may indicate that in movement disorders the processing of music is altered, which may in part related to the severity of motor deficits. An involvement of cerebellar structures in the pathogenesis of HD is well known.^[24]^

On the other hand both deficits might be linked by a deficit in perception and / or processing of rhythm, a function for which basal ganglia are know to be important.^[34][35]^ Affection of basal ganglia is an early event in HD, and thus, our results might also reflect a compensatory mechanism of the cerebellum in HD.

The results corroborate the importance of the cerebellum in music and especially rhythm processing.^[19][20][21][22]^ As the relations of cerebellar activation to motor performance scores were not only seen in simple movements (i.e. tapping), complex reaching movements (i.e. peg board) and general motor impairment (UHDRS), our results may be generalized. The fact that no relation was found with cognitive performance scores underlines the specificity of results. In contrast, manifest HDs activated less during listening to syllables compared to music. Furthermore, no correlation was observed between motor abilities and activity of the cerebellum, underlining the specific role of the cerebellum in extracting musical rhythmic structures in general and its relation to motor deficit severity in manifest HD in particular. 

Based one these findings one may hypothesize that music listening, making music and / or dancing may have beneficial effects in manifest HD, i.e. ameliorating motor dysfunction, which may be a question for future clinical studies. Lahav et al. pointed out, that motor performance can be enhanced even by passive listening to music.^[13]^ Several studies have shown that environmental enrichment has beneficial effects on cognitive and motor deficits in HD.^[36][37]^ Music itself has been used as a factor of environmental enrichment in animal models of other neurological disorders, where it improved cognitive functioning.^[38]^ Hence, one may hypothesize that music may have beneficial symptomatic or even disease modifying effects in HD, which may be a question for future clinical studies. 

## **Conclusion**

In the current we analyzed the modulation of music processing in an instance of a movement disorder, i.e. Huntington’s disease. Especially in manifestic HD alterations in music processing were observed, which were expressed by hyperactivations of cerebellar and medial prefrontal structures. Specifically, cerebellar hyperactivations were strongly related to the degree of movement dysfunction.    

## **Acknowledgements**

 We thank all participants.

## Address for correspondence: 

Carsten Saft, Department of Neurology, Huntington Centre NRW, St. Josef Hospital, Ruhr-University Bochum, Germany. Phone: +49-234-509-1, Fax: +49-234-509-2740, email: carsten.saft@ruhr-uni-bochum.de

*B. Pfleiderer and C. Saft contributed equally

## **Funding information**

This work was supported by the Ruhr-University Bochum (FoRUM grant 479/05).

## **Competing interests**

The authors have declared that no competing interests exist.
